# A Comprehensive Strategy to Pain Management for Cancer Patients in an Inpatient Rehabilitation Facility

**DOI:** 10.3389/fpain.2021.688511

**Published:** 2021-11-01

**Authors:** Terrence M. Pugh, Fabiana Squarize, Allison L. Kiser

**Affiliations:** ^1^Inpatient Oncology Rehabilitation, Atrium Health Carolinas Rehabilitation, Charlotte, NC, United States; ^2^Cancer Rehabilitation, Levine Cancer Institute, Charlotte, NC, United States; ^3^Atrium Health, Charlotte, NC, United States; ^4^Oncology Clinical Specialist, Atrium Health Carolinas Rehabilitation, Charlotte, NC, United States; ^5^Oncology Occupational Therapist, Atrium Health Carolinas Rehabilitation, Charlotte, NC, United States

**Keywords:** cancer, pain, inpatient rehabilitation facility, performance status, quality of life

## Abstract

Cancer pain has been shown to have a significant negative impact on health-related quality of life (HRQoL) for people experiencing it. This is also true for patients admitted to inpatient rehabilitation facilities (IRFs). An interdisciplinary approach is often needed to fully address a person's pain to help them attain maximum functional independence and to ensure a safe discharge home. Improving a patient's performance status in an IRF may also be a crucial determinant in their ability to continue receiving treatment for their cancer. However, if a person is determined to no longer be a candidate for aggressive, disease modulating treatment, IRFs can also be utilized to help patients and family's transition to comfort directed care with palliative or hospice services. This article will discuss the interventions of the multidisciplinary inpatient rehabilitation team to address a person's pain.

## Introduction

**Figure 1:**
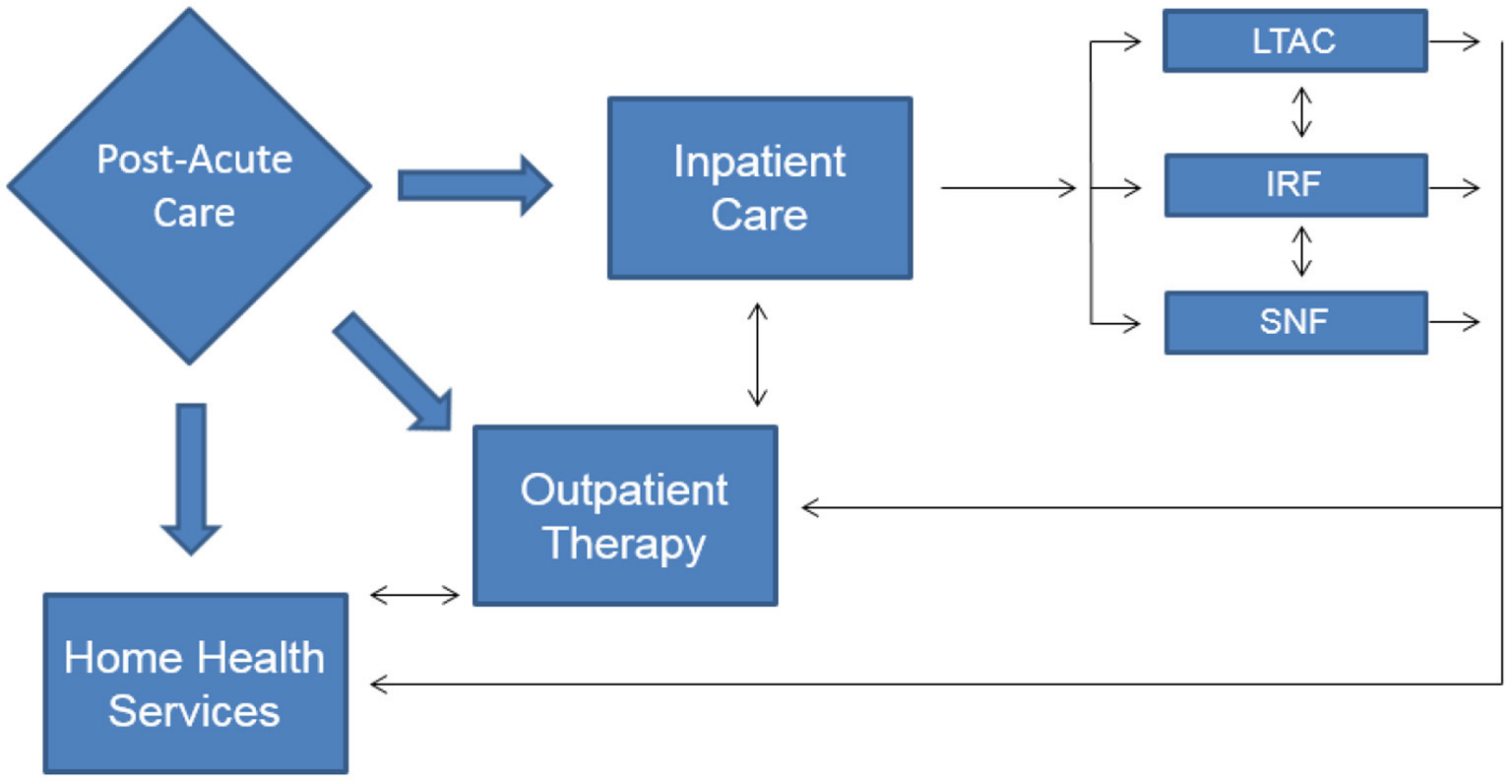
Post-acute care rehabilitation options.

There were over 1.8 million new cancer diagnoses in the United States (US) in 2020 (1) and as of January 2019, there were 16.9 million cancer survivors in the US with that number anticipated to grow to 22.2 million by the year 2030 (2). With the growing number of people alive with a cancer diagnosis, rehabilitation services are often utilized to address treatment-related or diagnosis specific impairments for patients across the post-acute care spectrum (Figure 1). The majority of cancer rehabilitation services are delivered in outpatient settings with only 2.4% of patients receiving inpatient rehabilitation services (3). Despite the relatively small number of patients accessing inpatient services, improvement in health-related quality of life (HRQoL) has been well-established when the rehabilitation team addresses impairments such as pain, fatigue, anxiety/depression and physical functioning (4). The following will provide an overview of the interventions that may be utilized in inpatient rehabilitation facilities to address and treat pain in the oncology patient. This framework can be tailored in a person-centered manner with considerations for medical diagnoses, patient perceptions and preferences in regard to the interventions listed.

## Medical Interventions

Identification of the type of pain experienced by the person is the first step in determining the appropriate intervention. There are several theories about the etiology of pain. Chronic pain (including cancer), inflammatory and neuropathic pain is a manifestation of neural plasticity in the peripheral (PNS) and central nervous system (CNS). This eventually leads to sensitization in both areas (5). Sensitization occurs due to chronic, hyperexcitability of sensory neurons in the PNS and an increase in the excitatory synapse transmission in the spinal cord, brain stem and cerebral cortex (5). Sensitization in the CNS has been shown to lead to long-term potentiation of pain in animal studies and is also a proposed mechanism in humans.

Fifty-six percentage of adults with cancer experience moderate to severe pain monthly (6) with 53% of people at all stages of cancer have pain (7). Pain can be further divided into more specific categories including acute (vs. chronic) pain, nociceptive (vs. neuropathic), migrainoid, post-operative, musculoskeletal/myofascial, osteoarthritic or cancer-related pain (8). Comprehensive pain management includes, pharmacologic, non-pharmacologic, integrative and interventional strategies (9). Clinicians should consider allergies, indications and contraindications for the recommended therapies prior to implementing a treatment plan.

### Opioids

**Figure 2:**
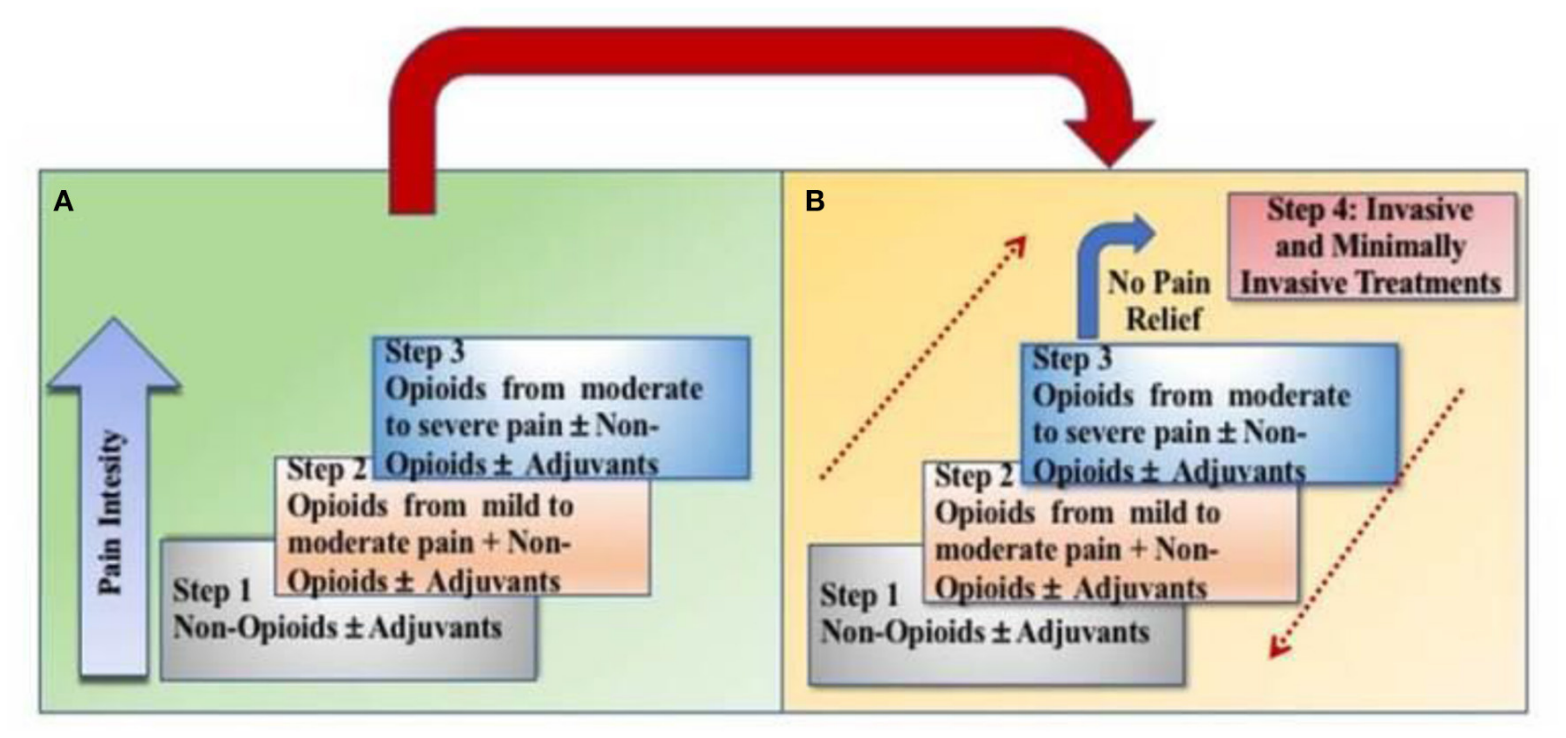
Transition from the original WHO three-step analgesic ladder **(A)** to the revised WHO fourth-step form **(B)**. The additional step 4 is an “interventional” step and includes invasive and minimally invasive techniques. This updated WHO ladder provides a bidirectional approach.

Cancer that is localized or metastasized is often very painful. The World Health Organization (WHO) pain ladder was originally designed in 1986 for patients experiencing cancer-related pain (10). The original ladder had three rungs (Figure 2). Early observational studies showed that when using this approach, 73% of people with cancer-related pain could achieve control (11). More recent data shows that strong opioids achieve a 75% response rate in reducing pain intensity by 3 points using a 0–10 scale (12). The goal of utilizing opioids in cancer pain management is to achieve adequate analgesia to improve function while minimizing adverse effects. In addition to the risk for addiction seen in other non-malignant, chronic pain patients, oncologic patients may also experience constipation, cognitive “clouding,” heartburn, nausea, hypogonadism, fatigue, infertility, osteoporosis/osteopenia, reduced libido, reduced frequency/duration or absence of menses, neurotoxicity, myoclonus, mood effects, increased risk for falls and new onset or worsening obstructive sleep apnea among others (13). To minimize the risks associated with chronic or high morphine milligram equivalent (MME) dosing, prescribers can use opioid conversion charts when switching between opioids (14). Risk of adverse events can also be mitigated by careful history taking and identification of risk factors including previous substance abuse or smoking. Pain agreements, pill counts, random drug testing and reviewing data from prescription drug monitoring programs can also be utilized to decrease the likelihood of misuse (13). Patient education about drug storage and disposal have also been shown to increase rates of safe opioid utilization (15).

### NSAIDs and Acetaminophen

For peri-operative pain a multi-modal approach including NSAIDs and acetaminophen has been recommended (8). For post-operative pain, NSAIDs (including cyclooxygenase-2 [COX-2] inhibitors) have also been shown to be effective. Celecoxib, a COX-2 inhibitor, dosed at 200 and 400 mg showed a 33 and 44% pain reduction, respectively, compared to placebo (16). Although there was concern about increased cardiac morbidity and mortality with utilization of celecoxib, a 2016 study showed non-inferiority of celecoxib to ibuprofen and naproxen in regard to cardiovascular safety for the treatment of arthritic pain (17). A single dose of ibuprofen was also shown to achieve 50% pain relief in half of patients with moderate to severe post-operative pain (18).

Acetaminophen is an option for mild to moderate pain and for fever reduction. At lower doses it is generally well-tolerated but does have hepatoxic potential and can even cause serum transaminase elevation (19). Close monitoring of liver function is recommended while on acetaminophen containing compounds. Patients taking acetaminophen should limit intake to <4,000 mg in a 24-h period.

### Neuropathic Pain

Chemotherapy-induced peripheral neuropathy (CIPN) is a clinical condition caused by neurotoxic medications used in the treatment of oncologic diagnoses. Common offending agents are taxanes, platinum-based compounds, vinca alkaloids, epothilones, eribulin, and bortezomib (20). CIPN not only impacts the nerves of the extremities causing pain, but also can cause throat discomfort, odynophagia and muscle cramps (19). Several classes of medications, included as adjuvants according to the WHO analgesic ladder, can be used to treat neuropathic pain. Tricyclic antidepressants (TCAs), serotonin norepinephrine reuptake inhibitors (SNRIs), anticonvulsants, topical analgesics, corticosteroids, bisphosphonates and cannabinoids have been used to treat painful neuropathy (21) (Table 1). Although a recent analysis showed that preventive medications, including acetyl-L-carnitine, should not be used to prevent CIPN. Treating oncologists can consider dose-reduced or alternative oncologic therapy. If a person develops painful neuropathy due to chemotherapy, then duloxetine should be offered (20). Topical treatment, dissolvable mucosal medications and injectable botulinum toxin have also been used to treat neuropathic pain. A 2015 review showed that topical lidocaine patches were safe and tolerable in people with neuropathic pain (22), however these studies showed efficacy that lasted <3 weeks duration (23). Topical capsaicin (patches or cream) may be effective in painful neuropathy with botulinum toxin type A also showing efficacy in peripheral neuropathic pain (22).

**Table 1:** Examples of neuropathic pain agents.

**Class**	**Medication examples**
Tricyclic antidepressants (TCAs)	Amitriptyline (Elavil®), Nortriptyline (Pamelor®)
Serotonin norepinephrine reuptake inhibitors (SNRIs)	Duloxetine (Cymbalta®), Venlafaxine (Effexor®)
Anticonvulsants	Gabapentin (Neurontin®), Pregabalin (Lyrica®)
Topicals	Capsaicin, Lidocaine (cream, Lidoderm® patch)

### Interventional Therapy

Recently a fourth rung was added to the WHO analgesic ladder and a bi-directional approach was added to include non-pharmacologic treatments for pain management including acute and chronic cancer pain. This allows for escalation and de-escalation of the pain management strategy as the person's clinical condition dictates. These include, but are not limited to, interventional procedures such as epidural or intrathecal analgesia, neuromodulation with or without a pump, nerve blocks or ablation procedures (23). Although these procedures may be beneficial for patients, interventional pain management is not often done in inpatient rehabilitation settings due to the need for prior authorization (PAs) and reimbursement limitations (24, 25). These should be considered in an outpatient setting if clinically indicated.

### Complementary Therapy

Acupuncture can be considered for patients with acute and chronic pain including cancer pain. This intervention is effective because sometimes cancer-related pain is difficult to classify as it is non-nociceptive and non-neuropathic (26). In certain animal models, electroacupuncture can release endogenous opioids from lymphocytes, monocytes, macrophages and granulocytes. This stimulation, in turn, suppresses pain signals in peripheral nerves (27). Electroacupuncture was shown to be effective in rats with both bone cancer and prostate cancer. This intervention was shown to decrease dynorphin and IL-1β to achieve analgesia (26). Inhibition of subfamily V member 1 protein through electroacupuncture was helpful in pain regulation in mice with carcinoma (28). In spite of these promising results, the use of acupuncture cannot be definitively recommended for the prevention of nor treatment of CIPN following the administration of neurotoxic chemotherapy (20). However, acupuncture and acupressure have been shown to be effective in reducing non-neuropathic cancer related pain and decreased use of analgesics (29).

Massage therapy has been shown to reduce cancer pain by ~40% (30). A recent meta-analysis showed that massage, including body massage, foot reflexology and aroma massage, was effective for cancer pain related to surgery, chemotherapy and metastatic disease. These effects were noted in all cancer types including breast and gastrointestinal malignancies (31). However, the effectiveness of massage therapy in managing cancer pain may also be influenced by other factors including the environment, music and aromas present during the session (32). Both acupuncture and massage therapy can be considered as adjunctive therapy if a qualified and licensed practitioner is available within the IRF.

## Therapy Interventions

In spite of aggressive medical management there are times that people with cancer pain require additional interventions to achieve pain control. A multidisciplinary team approach is utilized in inpatient rehabilitation facilities to address impairments, including pain, related to a patient's diagnosis and/or treatment. In addition to medical interventions, physical and occupational therapists along with rehabilitation nurses can assist people with cancer in managing their pain.

### Transcutaneous Electrical Nerve Stimulation

Transcutaneous electrical nerve stimulation (TENS) is a non-invasive modality that has been indicated to help with a variety of pain complaints, but most effective in neuropathic pain. The two most common types of TENS therapy are high frequency, low intensity TENS and acupuncture-like TENS (33). TENS works by utilizing the “gate theory” in which cells in the dorsal horn of the spinal cord are over stimulated thereby blocking pain signals to mitigate pain sensation (34). Although it may be beneficial for certain types of non-malignant pain, the data is inconclusive if it is beneficial for cancer-related pain (35). This should still be considered in patients with other non-malignant pain complaints. If TENS is utilized in a patient with an oncologic diagnosis, considerations must be made for contraindications including in areas overlying active tumor or in a person with unmanaged disease (36).

### Manual Therapy

Manual therapy (MT) is a complementary technique that uses precise hand techniques to improve muscular tissue restriction/mobilize soft tissue, relieve pain and to promote psychological well-being. Manual therapy can be performed by massage therapists, osteopathic medicine practitioners, chiropractors and physical therapists (37). Many people with cancer have utilized manual therapy to help with pain relief and to help improve well-being (38). A recent review showed that manual therapy was effective in managing cancer-related pain and improved overall feeling of wellness. More research is needed to determine if manual therapy can help with fatigue, anxiety, depression or nausea (36). Practitioners should use caution in insensate areas or in areas of vascular compromise. MT should be avoided in areas impacted by radiation dermatitis or over fragile bony areas due to metastatic disease or osteoporosis (35). Patients with myofascial pain syndrome are at risk of developing myofascial trigger points. In patients with pancreatic cancer, dry needling has been shown as an effective adjunct to neurolytic blocks for treatment of pain (39).

### Mindfulness Exercises

Mindfulness-based interventions have been used in the oncology population to reduce psychological distress. These interventions help reduce anxiety and depression, however improvement in other oncology specific impairments, including pain, have not been as well-studied (40). Cillessen et al. did show that mindfulness-based interventions did show improvement of oncologic impairments including fear of cancer recurrence, fatigue, sleep disturbance and pain (40). Mind-body exercise have been shown to positively impact bodily functions and improve pain by combining exercise with mental focus (41). Pilates have been shown to be effective in managing pain in patients with breast cancer (42). Yoga courses have also been utilized in the cancer rehabilitation population. Although yoga was shown to improve QoL, stress and fatigue in a recent analysis, improvement in pain was not shown (43). More research is needed for pilates and yoga before recommending as a part of a pain management plan for oncology patients (44).

### Other Interventions

Therapeutic touch can be described as using the hands on or near the body to help a patient. The non-invasive technique is used by nurses to aid in energy transfer to promote healing. This should be considered in oncology patients as it can reduce pain, anxiety and nausea in cancer patients (45). Heat and ice/cryotherapy can be used to manage pain in cancer patients, but should be used with caution over insensate areas or over areas with active or unmanaged disease (36). Both aroma therapy and music therapy have been shown to reduce pain in peri-operative breast cancer patients however more research is needed to recommend in other populations (46).

## Conclusion

Pain is a significant impairment that can limit a person's functional progress and cause psychological and physical distress while admitted to an inpatient rehabilitation facility. The rehabilitation team should consider a multi-modal, multidisciplinary pain intervention strategy to help a patient achieve control. Recommendations for treatment should be made on a case-by-case basis with considerations for type of cancer, disease burden, location and overall prognosis.

## Author Contributions

TP primary author and literature review. FS and AK consultants for interventions and manuscript review and editing. All authors contributed to the article and approved the submitted version.

## Conflict of Interest

The authors declare that the research was conducted in the absence of any commercial or financial relationships that could be construed as a potential conflict of interest.

## Publisher's Note

All claims expressed in this article are solely those of the authors and do not necessarily represent those of their affiliated organizations, or those of the publisher, the editors and the reviewers. Any product that may be evaluated in this article, or claim that may be made by its manufacturer, is not guaranteed or endorsed by the publisher.
